# Refractory Fetal and Neonatal Supraventricular Tachycardia Associated With Mitral Valve Mass

**DOI:** 10.7759/cureus.63944

**Published:** 2024-07-06

**Authors:** Marisa Pereira, Catarina Almeida, João Antunes Sarmento, Joana O. Miranda

**Affiliations:** 1 Pediatric Cardiology Department, Centro Hospitalar Universitário de São João, Porto, PRT; 2 Gynecology-Obstetrics and Pediatrics Department, Faculty of Medicine of the University of Porto, Porto, PRT; 3 Surgery and Physiology Department, Faculty of Medicine of the University of Porto, Porto, PRT

**Keywords:** cardiac hemangioma, cardiac tumor, accessory pathway, arrhythmia, supraventricular tachycardia

## Abstract

Primary cardiac tumors in children are rare and mostly benign but can cause significant cardiovascular complications, including arrhythmias.

We present a rare case of fetal and neonatal refractory supraventricular tachycardia linked to a probable mitral valve hemangioma, resulting in severe neonatal and maternal morbidity. Despite challenges, pharmacological therapy ultimately successfully managed the condition, highlighting the importance of individualized treatment in such complex cases.

## Introduction

Primary cardiac tumors in children are rare, occurring at an incidence of approximately 0.0017% to 0.28%, and are mostly benign. These tumors include rhabdomyomas, fibromas, and hemangiomas, among others. Despite their benign nature, cardiac tumors can lead to serious cardiovascular complications. One of the most significant complications is the development of arrhythmias, ranging from low-grade ectopy to life-threatening conditions such as supraventricular tachycardia, ventricular tachycardia, and sudden cardiac arrest [1-3].

Cardiac hemangiomas, a specific type of primary cardiac tumor, are vascular anomalies that are also rare and typically benign. They are often diagnosed incidentally due to their nonspecific presentation and can vary significantly in size and location within the heart. The clinical manifestations of cardiac hemangiomas are highly variable and depend largely on the tumor’s size and location. Symptoms may include arrhythmias, pericardial effusion, tamponade, congestive heart failure, right ventricular outflow tract obstruction, coronary insufficiency, and complete atrioventricular block. The natural history of cardiac hemangiomas is unpredictable; they may regress spontaneously, remain stable, or grow [4-6].

The precise mechanisms by which cardiac tumors, including hemangiomas, contribute to arrhythmias are not fully understood. Additionally, long-term outcomes for patients with cardiac hemangiomas and associated arrhythmias, particularly those diagnosed prenatally, are not well documented. Consequently, optimal management strategies for pediatric patients with cardiac tumors, especially those associated with severe arrhythmias, remain unclear.

The potential of cardiac tumors to exacerbate arrhythmias underscores the complexities of pharmacological management and the challenges in treating fetal and neonatal arrhythmias associated with these tumors. Furthermore, it highlights the potential for significant maternal and neonatal morbidity.

This article was previously presented as a meeting abstract and poster at the 56^th^ Annual Meeting of the Association for European Paediatric and Congenital Cardiology (AEPC) 2023, and it was also presented as a communication at the 2023 Portuguese Congress of Cardiology.

## Case presentation

**Figure 1:**
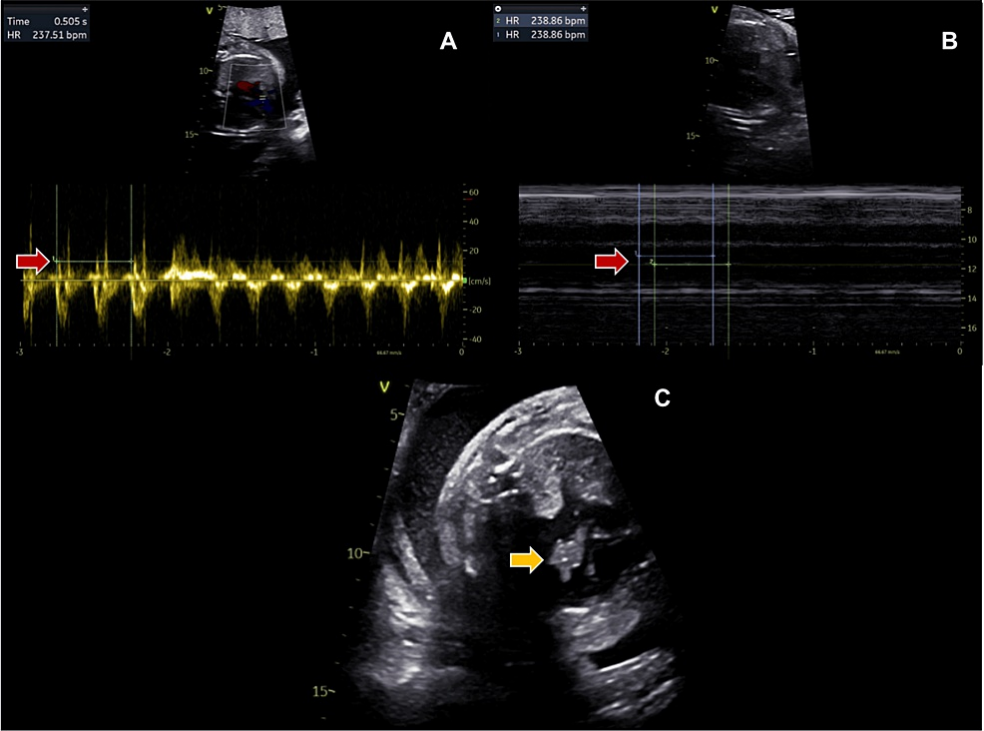
Fetal echocardiography image at 28 weeks of gestation. A) Color Doppler imaging captures blood flow through the fetal aortic valve, allowing for the measurement of the fetal heart rate. As indicated by the red arrow, in this case, it is 238 bpm. B) M-mode imaging slice captures the atrium and ventricle of the fetal heart, indicating a one-to-one relationship between atrial and ventricular contractions, showing normal electrical conduction from the atria to the ventricles (1:1 atrioventricular conduction). The heart rate is measured at 238 bpm, highlighted by the red arrow. These findings are consistent with supraventricular tachycardia. C) 2D imaging shows a homogeneous mass on the anterior leaflet of the mitral valve, measuring approximately 7x8 mm, indicated by the yellow arrow.

A pregnant woman was referred for fetal echocardiography at 28 weeks of gestation after tachycardia was observed during an obstetric ultrasound. Supraventricular tachycardia was identified, with an atrial rate of 220-240 bpm and 1:1 atrioventricular conduction (Figure 1). Additionally, a homogeneous mass measuring approximately 7x8 mm was found on the anterior leaflet of the mitral valve. This mass did not cause obstruction or regurgitation of the mitral valve flow. At this time, the fetus presented with hydrops fetalis, characterized by bilateral pleural effusion and ascites. Transplacental treatment was initiated, consisting of intravenous digoxin and oral flecainide, following our institution’s protocol.

After 14 days of treatment, at 30 weeks of gestation, fetal arrhythmia persisted, albeit with a slightly lower atrial rate (180-190 bpm). The mitral valve tumor, though apparently larger (measuring 19x12 mm), did not cause any intracardiac flow disturbances. Due to the progression of hydrops fetalis and maternal drug toxicity signs (frequent nausea and vomiting, elevated liver enzymes, first-degree atrioventricular block, and nonspecific changes in ventricular repolarization), digoxin was discontinued, and oral amiodarone was initiated.

However, after six hours of amiodarone therapy, the analytical study revealed worsened maternal pharmacological toxicity, characterized by a significant increase in liver enzymes and changes in thyroid function. It was then decided to terminate the pregnancy via cesarean section, taking into account that, at that moment, the maternal risks were considered higher than the potential benefits of continuing fetal therapy.

**Figure 2:**
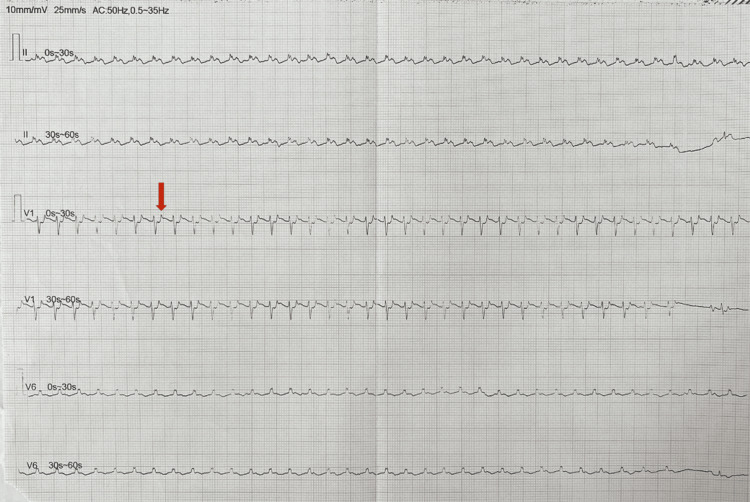
Electrocardiogram after birth. Supraventricular tachycardia with an atrial rate of approximately 200 bpm, a retrograde P wave (red arrow), and a short RP interval.

**Figure 3:**
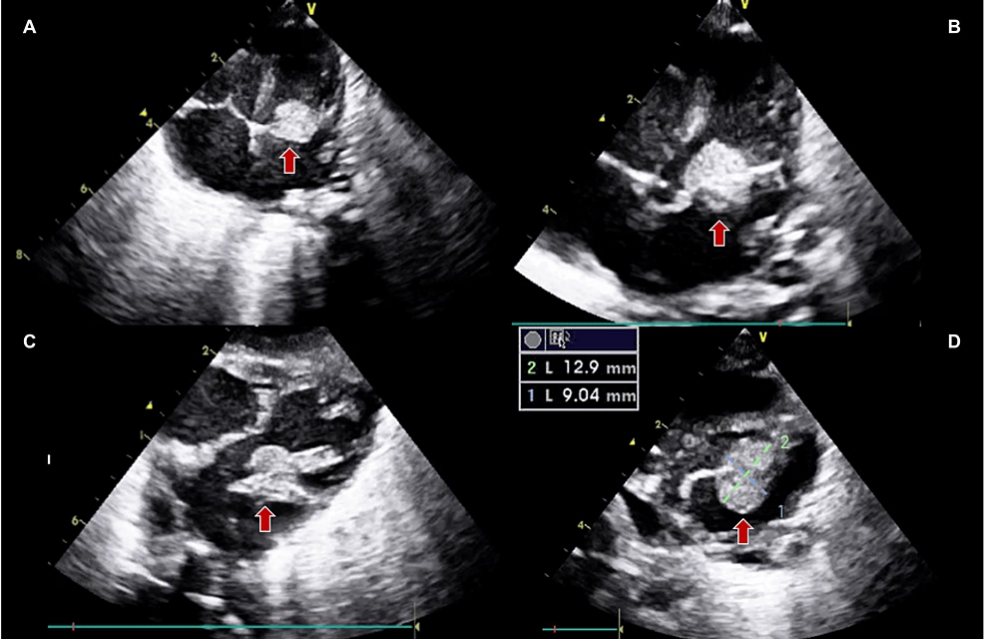
Transthoracic echocardiography after birth. 2D imaging of the 4-chamber view (A) and the 5-chamber views (B and C) shows a homogeneous mass (red arrow) on the anterior leaflet of the mitral valve, measuring approximately 13x9 mm (D).

A premature female newborn weighing 2000 grams was then born with an initial APGAR score of 5, necessitating positive pressure ventilation, endotracheal intubation, and paracentesis due to significant ascites. The electrocardiogram (Figure 2) revealed supraventricular tachycardia with an atrial rate of approximately 200 bpm, retrograde P waves, and a short RP interval. Once the patient was hemodynamically stable, pharmacological cardioversion with adenosine was attempted, with reversion to sinus rhythm but rapid re-entry on supraventricular tachycardia. Subsequently, an amiodarone infusion was initiated, starting with a loading dose of 5 mg/kg over 60 minutes, followed by a maintenance dose of 6 mcg/kg/minute, although sustained reversion to sinus rhythm was not achieved. At this time, the transthoracic echocardiogram confirmed a mass on the anterior leaflet of the mitral valve, measuring approximately 13x9 mm, without causing obstruction or regurgitation of the mitral flow (Figure 3).

On day two of life, there was an episode of wide complex tachycardia (interpreted as supraventricular tachycardia with aberrant conduction), leading to an increase in the maintenance dose of amiodarone to 10 mcg/kg/minute. Subsequently, due to the patient's development of hemodynamic instability, synchronized electrical cardioversion was attempted. However, neither chemical nor electrical cardioversion resulted in sustained rhythm control. Therefore, lidocaine infusion was initiated, consisting of a bolus loading dose of 1 mg/kg, followed by a maintenance dose of 20 mcg/kg/minute. This intervention significantly impacted the patient's rhythm, resulting in progressively longer periods of sinus rhythm and hemodynamic stability.

On day nine of life, while on amiodarone and lidocaine, sustained sinus rhythm was achieved. At this point, due to the potential side effects and long half-life of amiodarone, both amiodarone and lidocaine were discontinued, and oral propranolol was initiated at a dosage of 1 mg/kg every eight hours.

After achieving sustained hemodynamic stability, a complementary abdominal ultrasound was performed on day 18 of life, revealing a 12 mm hyperechogenic mass on hepatic segment IVa, suggestive of a hemangioma.

**Figure 4:**
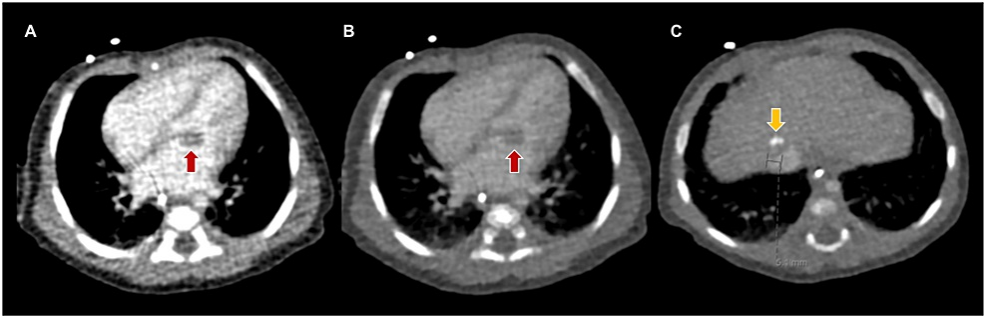
Cardiac and abdominal computed tomography image. Cardiac (A and B) and abdominal (C) computed tomography images show masses in both the heart (red arrow) and liver (yellow arrow), which exhibit very similar imaging characteristics: both are vascularized with intense contrast agent uptake and both contain calcification foci.

Subsequently, cardiac and abdominal computed tomography scans were conducted on day 23 of life to further characterize both masses (Figure 4). These tumors exhibited very similar imaging characteristics, including intense contrast agent uptake and the presence of calcification foci. Their similar evolution patterns suggested a potential shared histological origin. The hepatic lesion displayed imaging diagnostic hallmarks of a congenital hemangioma, a diagnosis further supported by its progression to complete calcification and subsequent resolution. Likewise, the cardiac lesion also exhibited progressive calcification and stabilization in size, coinciding with the improvement in heart rhythm.

A Next Generation Sequencing panel for RASopathies was conducted but did not identify any pathogenic variants.

Although a definitive histological diagnosis could not be obtained, the similarities in imaging characteristics and clinical progression of both tumors led to the assumption of congenital hemangiomas affecting both the cardiac and hepatic regions.

Between day nine and day 37, several beta-blocker administrations were skipped due to episodes of sinus bradycardia related to oral propranolol. On day 37, premature atrial contractions were recorded, with increasing frequency until day 42 when sustained supraventricular tachycardia was detected. Once again, there was no sustained reversion to sinus rhythm after pharmacological cardioversion with adenosine and amiodarone infusion (a loading dose of 5 mg/kg over 60 minutes, followed by increasing maintenance doses up to a maximum of 9 mcg/kg/minute).

A new synchronized electrical cardioversion was performed due to hemodynamic instability, but it also failed to revert to sinus rhythm. Lidocaine infusion was again initiated (with a loading dose of 1 mg/kg, followed by increasing maintenance doses up to a maximum of 35 mcg/kg/minute). After reintroducing lidocaine, alternating periods of sinus rhythm and supraventricular tachycardia occurred, with the patient achieving sustained sinus rhythm since day 53 of life.

At this point, amiodarone and lidocaine were once again discontinued, and the patient was started on both oral propranolol (1 mg/kg every eight hours) and flecainide (maximal dose of 4 mg/kg/day every 12 hours). She was discharged from the hospital on day 67 of life, maintaining both oral antiarrhythmic drugs.

Currently, after one year of follow-up, the patient continues to take oral propranolol (1 mg/kg/dose every eight hours) and oral flecainide (3.5 mg/kg/day every 12 hours). Throughout the serial electrocardiograms and 24-hour Holter monitoring conducted so far, the patient consistently exhibits sinus rhythm, along with first-degree atrioventricular block (likely iatrogenic), a normal corrected QTc interval, no ventricular pre-excitation, and no documented supraventricular tachycardia episodes. On the echocardiogram, the mitral valve mass involuted and became less significant relative to the size of the heart.

## Discussion

We report a rare and challenging case of fetal and neonatal refractory supraventricular tachycardia associated with a probable mitral valve hemangioma, resulting in significant maternal and neonatal morbidity.

Primary cardiac tumors can be associated with serious cardiovascular complications, including clinically significant arrhythmias. The optimal approach for patients with cardiac tumors remains unclear, especially when severe arrhythmias are present. Cardiac hemangiomas, in particular, can be associated with a variety of symptoms depending on the tumor's location, which can include arrhythmias [1-6].

Historical reports of cardiac tumors primarily focus on benign outcomes and incidental findings. In contrast, our case illustrates the significant clinical impact of a mitral valve hemangioma, contributing to severe supraventricular tachycardia and necessitating aggressive pharmacological intervention. This case uniquely presents both fetal and neonatal refractory supraventricular tachycardia, highlighting the continuity and severity of the arrhythmia across different developmental stages.

Unlike many previous cases where initial pharmacological therapy led to sustained rhythm control, our patient required multiple agents and adjustments due to persistent arrhythmias and maternal drug toxicity. The therapeutic challenge and detailed management strategy provide new insights into handling similar cases. The case also emphasizes the significant maternal morbidity due to drug toxicity and the difficult decision-making process leading to premature cesarean delivery, highlighting the need for a careful balance between maternal and fetal risks in managing such cases.

Additionally, the presence of a concurrent hepatic hemangioma presents a novel aspect of multi-organ involvement, requiring comprehensive diagnostic and therapeutic approaches. This adds a layer of complexity to the management and follow-up of the patient.

In infancy, supraventricular tachycardia predominantly results from accessory pathways. In this particular case, the reversion to sinus rhythm with the administration of adenosine, although not sustained, makes probable the hypothesis of atrioventricular re-entry tachycardia via a concealed accessory pathway. The mitral valve tumor may have acted as a trigger for the tachycardia episodes, working synergistically and contributing to rhythm instability, making management challenging [7-10].

This case underscores the importance of individualized treatment strategies in managing complex cardiac tumors associated with arrhythmias. It highlights the need for vigilant monitoring of maternal and neonatal conditions, prompt recognition of drug toxicity, and multidisciplinary collaboration in decision-making. The significant maternal and neonatal morbidity in this case, including forced premature birth and pharmacological side effects such as hypothyroidism, underscores the complications inherent in managing such conditions.

While this case provides valuable insights, it is limited by the absence of histological confirmation of the cardiac and hepatic masses as hemangiomas. Future studies should focus on genetic testing to elucidate underlying etiologies and refine treatment protocols for similar challenging cases.

In summary, this case of refractory supraventricular tachycardia associated with a mitral valve hemangioma highlights the complexities and individual nuances in managing pediatric patients with cardiac tumors. By contrasting with historical reports, we underscored critical differences and provided new insights into the management and outcomes of such cases, contributing novel information to the medical community.

## Conclusions

Managing cases like ours presents a complex and multifaceted challenge. Although clinical guidelines are published, each case reveals unique aspects that increase the complexity of both diagnosis and treatment, often challenging even the most up-to-date protocols. Therefore, effective management necessitates not only familiarity with existing guidelines but also personalized assessment and careful adaptation of treatment strategies tailored to each patient's unique clinical presentation and response to therapy.

Our experience underscores the critical importance of individualized approaches in navigating the complexities of fetal and neonatal refractory supraventricular tachycardia associated with cardiac tumors. This includes balancing aggressive pharmacological interventions with vigilant monitoring for maternal and neonatal well-being, as highlighted by our case involving a probable mitral valve hemangioma. Furthermore, the concurrent identification of a hepatic hemangioma adds a novel dimension, emphasizing the need for comprehensive diagnostic evaluation and multidisciplinary collaboration.

Moving forward, ongoing research and advancements in genetic testing hold promise for elucidating underlying etiologies and refining treatment protocols. By sharing our insights and contrasting them with historical reports, we aim to contribute to improved understanding and optimized outcomes for similar complex cases in pediatric cardiology.
